# Optical Coherence Tomography and Magnetic Resonance Imaging in Multiple Sclerosis and Neuromyelitis Optica Spectrum Disorder

**DOI:** 10.3390/ijms17111894

**Published:** 2016-11-15

**Authors:** Praveena Manogaran, James V. M. Hanson, Elisabeth D. Olbert, Christine Egger, Carla Wicki, Christina Gerth-Kahlert, Klara Landau, Sven Schippling

**Affiliations:** 1 Neuroimmunology and Multiple Sclerosis Research, Department of Neurology, University Hospital Zürich and University of Zurich, Frauenklinikstrasse 26, 8091 Zurich, Switzerland; praveena.manogaran@usz.ch (P.M.); james.hanson@usz.ch (J.V.M.H.); elisabethdaniela.olbert@usz.ch (E.D.O.); christine.egger@usz.ch (C.E.); carla.wicki@usz.ch (C.W.); 2 Department of Ophthalmology, University Hospital Zurich and University of Zurich, Frauenklinikstrasse 24, 8091 Zurich, Switzerland; christina.gerth-kahlert@usz.ch (C.G.-K.); klara.landau@usz.ch (K.L.)

**Keywords:** multiple sclerosis, neuromyelitis optica spectrum disorder, optical coherence tomography, magnetic resonance imaging, optic neuritis, visual pathway, optic nerve, optic radiations, visual cortex

## Abstract

Irreversible disability in multiple sclerosis (MS) and neuromyelitis optica spectrum disorder (NMOSD) is largely attributed to neuronal and axonal degeneration, which, along with inflammation, is one of the major pathological hallmarks of these diseases. Optical coherence tomography (OCT) is a non-invasive imaging tool that has been used in MS, NMOSD, and other diseases to quantify damage to the retina, including the ganglion cells and their axons. The fact that these are the only unmyelinated axons within the central nervous system (CNS) renders the afferent visual pathway an ideal model for studying axonal and neuronal degeneration in neurodegenerative diseases. Structural magnetic resonance imaging (MRI) can be used to obtain anatomical information about the CNS and to quantify evolving pathology in MS and NMOSD, both globally and in specific regions of the visual pathway including the optic nerve, optic radiations and visual cortex. Therefore, correlations between brain or optic nerve abnormalities on MRI, and retinal pathology using OCT, may shed light on how damage to one part of the CNS can affect others. In addition, these imaging techniques can help identify important differences between MS and NMOSD such as disease-specific damage to the visual pathway, trans-synaptic degeneration, or pathological changes independent of the underlying disease process. This review focuses on the current knowledge of the role of the visual pathway using OCT and MRI in patients with MS and NMOSD. Emphasis is placed on studies that employ both MRI and OCT to investigate damage to the visual system in these diseases.

## 1. Introduction

Multiple sclerosis (MS) is a chronic autoimmune inflammatory disorder of the central nervous system (CNS) in which different environmental factors act on the basis of a multi-genetic trait [1,2]. It is characterized by focal demyelinating plaques and diffuse neurodegeneration throughout the white and gray matter [3,4]. Approximately two million people worldwide are affected by this disorder, and it is the most common non-traumatic neurological disability affecting young adults [5]. Neuromyelitis optica spectrum disorder (NMOSD) is a rare autoimmune astrocytopathic disease of the CNS that preferentially involves the optic nerve and spinal cord [6,7]. The identification of the disease-specific NMO-immunoglobulin G (NMO-IgG) and its effects on astrocytic aquaporin-4 (AQP4) water channels has helped to facilitate differentiation of NMOSD from MS [8]. Nevertheless, imaging and clinical manifestations frequently overlap, particularly in the early stages of the diseases and in NMOSD patients testing negative for AQP4-IgG antibodies [7]. The exact pathophysiological mechanisms behind MS and NMOSD have still not been fully elucidated; however, there is evidence that tissue injury and demyelination in MS is mediated by T-cell activity [9]. Axonal and neuronal atrophy is likely a secondary effect of inflammatory demyelination but can also occur as a result of independent subclinical disease activity [9]. Neuro-axonal degeneration (in addition to demyelination) has recently been considered more relevant in MS pathophysiology; it has been documented in both active and inactive lesions, distal to the areas affected by autoimmune inflammation, and early in the disease course [10]. Conversely, the pathophysiology in NMOSD predominantly involves the deposition of IgG and complement, resulting in a loss of AQP4 proteins on astrocytes and severe neuronal and axonal loss [6].

Both diseases present with significant clinical and pathological heterogeneity between individuals. However, visual impairment appears to be prominent in both disorders, with acute optic neuritis (ON) frequently occurring as the initial symptom [11,12]. ON is an inflammation of the optic nerve that is usually accompanied by pain on eye movement followed by visual loss [13]. Recovery of visual function is often incomplete, likely due to persistent demyelination and—in more severe cases—axonal loss [13]. ON is typically more severe, recurrent, and frequently bilateral in NMOSD compared to MS [14,15].

Optical coherence tomography (OCT) is a safe and non-invasive 3D imaging tool that uses low coherent, near-infrared light to generate cross-sectional images of the retina, which can be used to quantify axonal and neuronal atrophy [16]. Three-dimensional volumetric scans can be formed from multiple cross-sectional images of the retina and, with contemporary spectral-domain OCT (SD-OCT) devices, identification and delineation (”segmentation”) of the individual layers is now possible. SD-OCT is more reproducible, faster and has better resolution than previous time-domain (TD-OCT) devices [17].

**Figure 1:**
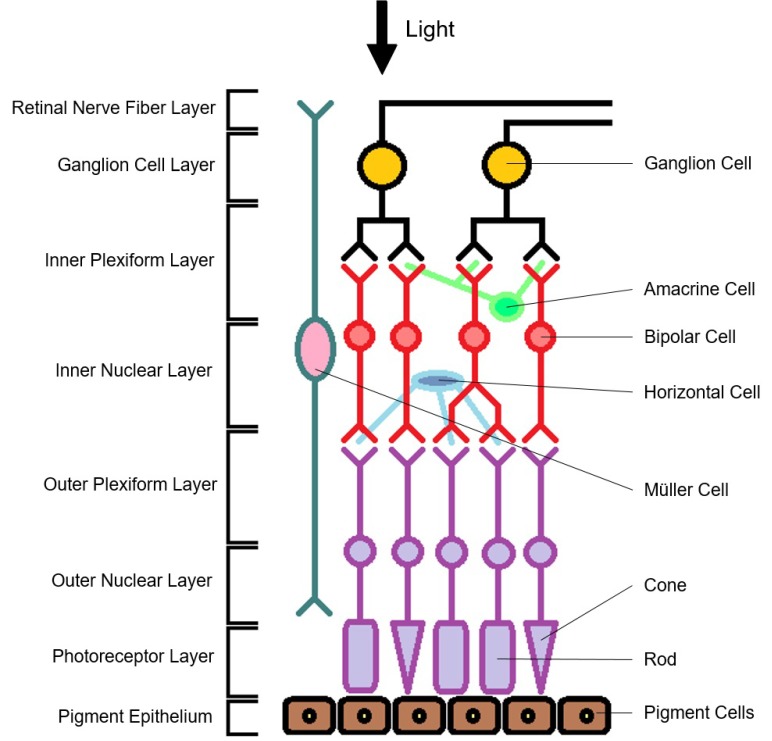
The different layers and cells that make up the retina. Retinal nerve fiber layer (RNFL), ganglion cell layer (GCL), inner plexiform layer (IPL), inner nuclear layer (INL), outer plexiform layer (OPL), and outer nuclear layer (ONL), and photoreceptor layer (PR).

When examining the retina, numerous studies have demonstrated peripapillary retinal nerve fiber layer (RNFL) thinning and decreased total macular volume (TMV) in NMOSD and MS, and this thinning is more prominent in patients with a history of ON but is also observed in those without [18,19,20,21]. RNFL thinning reflects damage to unmyelinated ganglion cell axons and may be due to ON-related retrograde degeneration (damage propagating backwards from the site of injury). Interestingly, structural damage to the retina can occur with or without the presence of ON in both MS and NMOSD. A previous study found thinning of the ganglion cell layer (GCL) and inner plexiform layer (IPL) in patients with clinically isolated syndrome (CIS), suggesting that not only is neurodegeneration a prominent component of MS pathology, but that it can occur early in the disease course [22]. The damage seen in eyes without a history of ON is likely due to subclinical disease activity, which is commonly identified in MS but has only recently been reported in NMOSD. It is possible that in NMOSD, Müller cells (which span the entire thickness of the retina and contain an abundance of AQP4 channels; Figure 1) may be affected in patients without ON history [23], although this remains unconfirmed [20]. Moreover, lesions in other parts of the visual pathway may have affected the retina via retrograde degeneration. Macular RNFL is less commonly studied compared to peripapillary RNFL but appears to be thinner in MS patients with or without a history of ON compared to healthy controls [24].

GCL and IPL thinning have been observed in both MS and NMOSD; in particular, NMOSD patients typically exhibit thinner GCL and IPL compared to MS patients [23,25], most likely reflecting more severe neuronal and axonal involvement in NMOSD [26]. It is likely that retrograde degeneration begins at the ganglion cell axons (RNFL), progresses to the cell bodies (located in the GCL) and then to the dendrites (located in the IPL), where degeneration appears to stop (Figure 1) [25,27].

Inner nuclear layer (INL) thickness is associated with inflammatory disease activity in MS [28]. While INL thinning has been documented in a minority of MS patients [29], INL thickening has been observed more frequently [20,30,31] and seems to have the potential to differentiate between MS and NMOSD [24]. INL thickening is often associated with microcystic macular edema (MME) [20,31] but can also occur independently of MME [30]. MME is the finding of intra-retinal cyst-like spaces in the INL [32,33]. It is thought to result from the breakdown of the blood-retinal barrier with or without microglial inflammation and subsequent accumulation of fluid within the retina [33], but it has also been suggested to be due to impaired water or potassium homeostasis [32]. The outer nuclear layer (ONL) contains the cell bodies of rods and cones; previous studies have found no significant differences in ONL thickness between healthy controls and MS [25,34] or NMOSD [20] patients. 

Magnetic resonance imaging (MRI) is a valuable tool used for identifying, characterizing and quantifying CNS pathology in MS and NMOSD [35]. It is a reproducible and sensitive method with the advantage of being able to obtain measurements from the entire brain and spinal column. Unlike in MS, NMOSD was previously thought to feature no brain involvement; however, it is now well-established that development of non-specific brain lesions can also occur in NMOSD [36].

Conventional imaging techniques are commonly used to monitor disease activity by quantifying lesion load (lesion number and volume on T2- or gadolinium enhanced T1-weighted images) and, more recently, neurodegeneration, through the measurement of global brain volume loss (based on anatomical T1 and T2/FLAIR data), frequently referred to as brain atrophy. Brain volume loss can be assessed either cross-sectionally, using brain parenchymal fraction (BPF), or longitudinally, using registration-based approaches such as SIENA (Structural Image Evaluation, using Normalization, of Atrophy) [37], to reflect tissue atrophy caused by both demyelination and axonal loss [38,39,40]. While lesion detection and qualitative assessments with conventional imaging techniques are used in clinical practice for diagnosis and monitoring, this approach lacks pathological specificity and is therefore unsuitable for characterizing the biological mechanisms and temporal sequences underlying inflammatory demyelination and neuro-axonal degeneration in MS and NMOSD [41]. Further difficulty arises from the fact that the complex pathological processes largely differ between MS and NMOSD in a manner that has yet to be fully characterized [42,43]. 

In contrast, advanced MRI techniques may provide a more quantitative and potentially more sensitive tool to detect subtle changes in the normal appearing white or gray matter (NAWM/NAGM), which are often undetectable by conventional MRI sequences [43]. Magnetic resonance spectroscopy (MRS), for example, can record signals from metabolites like *N*-acetylaspartate (NAA), which is associated with axonal integrity and demyelination [44,45]. Myelin water imaging (MWI) and magnetization transfer imaging (MTI) provide a sensitive in vivo measurement of myelin water and macromolecule content within the CNS, respectively, whilst diffusion tensor imaging (DTI) can be used to examine the integrity of the neural tracts [46,47,48]. DTI is the most commonly used technique for examining the visual pathway, particularly the optic nerves, chiasm and optic radiations [49,50]. Nevertheless, both conventional and advanced MRI techniques can provide complementary information about the visual pathway.

OCT and MRI can be used to reliably monitor neurodegeneration in experimental models of MS such as myelin oligodendrocyte glycoprotein (MOG) peptide induced experimental autoimmune encephalomyelitis (EAE), a common MS model in rodents. Retinal changes have been detected longitudinally with OCT and histo-morphological measurements, both before clinical manifestation, and also during the disease course in MOG induced EAE rats [51]. Deterioration of the RNFL, GCL and IPL has similarly been observed in MOG-EAE mice with ON, indicating a significant loss of retinal ganglion cells over time [52]. Additionally, the visual pathway is selectively affected in MOG-EAE mice, with both axonal and myelin injury observed using diffusion MRI and immunohistochemistry [53,54,55]. Studies combining OCT and MRI as tools for investigating the visual system in murine models of MS are scarce, and have yet to be conducted with SD-OCT [56]. Therefore, this review will primarily focus on human studies involving OCT and MRI.

Researchers have recently begun to explore the relationship between OCT-derived measures of retinal layer thickness changes and MRI-derived brain volume loss in order to understand the propagation of neuronal and axonal dysfunction throughout the CNS. These tools can be used to study how damage to one part of the CNS may affect others, and to investigate the role of retrograde and anterograde degeneration in the visual pathway. The objective of this manuscript is to provide an extensive review of the current literature examining the relationships between OCT and MRI measures in both MS and NMOSD.

## 2. Global Brain Measurements

**Figure 2:**
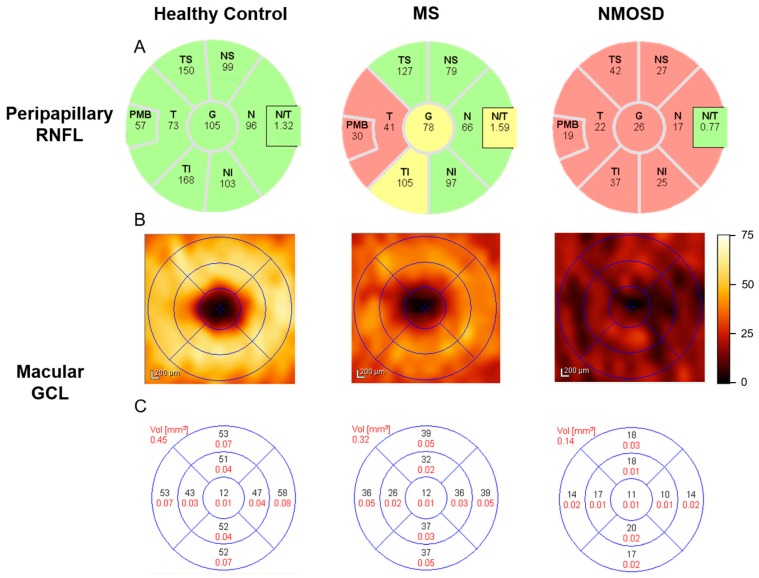
OCT-derived peripapillary RNFL and macular GCL thickness (μm) for the right eye of a healthy control, MS and NMOSD patients. (**A**) Peripapillary RNFL average thickness (µm) for each quadrant. Green = RNFL thickness between the 95th and 5th percentile of a built-in normative database, Yellow = RNFL thickness between the 5th and 1st percentile, Red = RNFL thickness less than the 1st percentile; (**B**) Macular GCL thickness (µm) map; and (**C**) macular GCL average thickness (µm; in **black**) and volume (mm^3^; in **red**) for each quadrant. OCT = optical coherence tomography, RNFL = retinal nerve fiber layer, GCL = ganglion cell layer, MS = multiple sclerosis, NMOSD = neuromyelitis optica spectrum disorder, T = temporal, S = superior, N = nasal, I = inferior, G = global, PMB = papillomacular bundle, N/T = nasal:temporal ratio.

A positive correlation between BPF, RNFL thickness and TMV was observed in a cohort of MS patients with and without a history of ON [40,57]. This relationship suggests the presence of widespread neurodegeneration in the CNS of MS patients, including the brain and optic nerve (Figure 2). Another index of brain atrophy, bicaudate ratio, was also correlated with RNFL thickness in MS patients [58]. Furthermore, a prospective study in early MS found an inverse correlation with BPF and white matter fraction—but not with gray matter fraction—compared to RNFL thickness and TMV [10]. The existing gray matter association appeared to be due to an age-related effect, suggesting that gray matter atrophy may not be prominent in early MS [10]. This is further supported by data from MS patients with greater disability and longer disease duration, in whom an association between RNFL and normalized gray and white matter volume in eyes with or without ON history was found [59]. Interestingly, there was an association between normalized brain, gray matter and white matter volume and TMV, in MS eyes without a history of ON, but only in normalized white matter volume for eyes with a history of ON [59]. The apparent discrepancy with regard to TMV may be because TMV reflects the integrity of numerous distinct layers of the retina, both neuronal and axonal, and as such can only provide a non-specific measure of retinal atrophy.

In MS eyes with and without a history of ON, there was a correlation between normalized brain volume, white matter volume and GCL [59]. Other studies found that the peripapillary RNFL, GCL-IPL, and INL thickness was associated with normalized gray and white matter volumes only in MS patients without prior ON history [60,61]. In many studies, GCL and IPL are frequently aggregated for analysis (GCL-IPL) due to difficulty in accurately distinguishing the two layers visually and with OCT segmentation tools [62]. Retinal damage seen outside the context of ON history may be due to the diffuse effects of the disease throughout the CNS or due to atrophy in other parts of the visual system (via retrograde degeneration). This also suggests that gray matter in the CNS is comparable to the neuronal components of the retinal GCL [25,61]. However, it is possible that gray matter relationships are clouded by the presence of white matter atrophy caused by ON. In primary progressive MS (PPMS), peripapillary RNFL thickness was associated with NAWM volume [61]. Interestingly, there was no relationship between RNFL thickness and NAA concentration, measured with MRS in PPMS NAWM [57]. The relationships seen with global atrophy measures raise the possibility that trans-synaptic degeneration is not restricted to the visual pathway, but is rather a pathological phenomenon that disseminates throughout the MS brain.

An increased rate of thinning in GCL-IPL was correlated with increased rate of cortical gray, cortical white, or whole brain atrophy [28]. The relationship between cerebral volume fraction (a measure of whole brain atrophy) and the rate of GCL-IPL thinning was seen in all the MS subtypes, however, a stronger relationship was seen in progressive MS subtypes compared to relapsing-remitting MS (RRMS) [28]. Additionally, the rate of change in GCL-IPL thickness was related to the rate of fluid-attenuated inversion recovery (FLAIR)-lesion volume accumulation in RRMS patients [28]. The rate of thinning in GCL-IPL appears to reflect global neurodegeneration and is linked to new disease activity or disease progression; it may also be superior as a method of tracking degeneration over time compared to the rate of thinning in the peripapillary RNFL [28].

RNFL and TMV did not correlate with either T2 hyper-intense lesion volume or T1 hypo-intense lesion (‘black hole’) volume, both of which are non-specific markers for disease activity in MS [10,63]. In RRMS, INL thickness in eyes without prior ON was also associated with lesion volume [61]. The association between these OCT measures and lesion volume suggests that the mechanisms involved in retinal pathology of these layers may be primarily inflammatory [61]. Additionally, the rate of change in GCL-IPL and INL thickness was related to the rate of lesion volume accumulation in RRMS patients [28]. Interestingly, the same authors had previously found that increased baseline INL thickness was associated with a greater chance of developing new lesions and also predicted the development of relapses and disability progression in MS [64]. They suggested that the observed increase in INL thickness seen in previous studies might represent inflammatory activity in MS and that INL thickness would decline over time [28]. Monitoring INL changes in RRMS may provide important information on global inflammation longitudinally. 

Intracranial volume (ICV) was correlated with GCL-IPL and ONL but not with INL, peripapillary or macular RNFL thickness in MS [61]. There was a relationship observed in healthy controls for all three OCT measures compared to ICV [61]. The authors suggest that this provides support for a biological relationship between ICV and OCT thickness measures [61].

A lack of standardization in terms of statistical analysis and OCT scan protocols makes it difficult to compare results between studies. In particular, potential concerns arise when comparing OCT results with MRI findings. As visual information from each retina is conveyed to both sides of the brain, it is currently not possible to correlate whole-brain-derived MRI data with OCT data from individual eyes. An additional consideration is that both eyes of an individual patient are correlated to some degree, and therefore consideration of intra-patient inter-eye dependencies is vital when analyzing or modelling OCT results [10].

## 3. Optic Nerve

The optic nerve is a frequent site of injury in both MS and NMOSD and can be used to study both axonal and neuronal degeneration in these diseases. When comparing OCT data to measures of atrophy in the optic nerve obtained with fast spin echo MRI, a strong positive correlation was seen with peripapillary RNFL thickness in MS patients [65]. A decrease in both the optic nerve lesion magnetization transfer ratio (MTR) and entire optic nerve MTR correlated with RNFL thinning and reduced TMV in a cohort including early MS and CIS patients [66]. Similarly, abnormal DTI was positively associated with RNFL thinning in the optic nerves of patients with MS [49,67], in MS patients with a history of ON [67] and also in a cohort including MS, NMOSD, and CIS [68]. This suggests that axonal loss may be a pathological contributor to both MTI and DTI abnormalities. In a longitudinal DTI study, reduced axial diffusivity (evidence of axonal fragmentation) in the optic nerve one month after ON was positively correlated with RNFL thickness in the affected eyes of CIS and early MS patients at both 6 and 12 months after ON [69]. This indicates that axial diffusivity of the optic nerve may be predictive of axonal damage in the retina that occurs in the months after an ON episode [69]. During the chronic phase of NMOSD, peripapillary RNFL was significantly associated with ON lesion length [70]. Acute inflammatory lesions can result in physical or functional axonal damage to fibers passing through the optic nerve lesions and, via retrograde degeneration, axonal loss could continue onto distal regions such as the retina.

A negative correlation was observed between GCL-IPL thickness and the length of optic nerve lesions one to two months following the onset of ON episodes [71]. These results suggest that atrophy of the retinal ganglion cells may be greater in cases with longer lesions in the optic nerve [71]. However, there was no relationship between proximity of lesions to the retina and GCL-IPL thinning, which could suggest the involvement of factors other than retrograde degeneration [71]. 

TMV is also positively correlated with the area of the optic nerve lesions in MS patients [65] and with DTI measures of axial and radial diffusivity in MS patients without a history of ON [67]. Interestingly, in MS patients with a history of ON, TMV was correlated with increased radial diffusivity (an indicator of demyelination) but not with axial diffusivity [67]. The relationship between decreased TMV and optic nerve damage is thought to be due to retrograde degeneration extending throughout the entire retina [65].

## 4. Optic Tract

The optic tract is the post-chiasmal, pre-geniculate part of the visual pathway that includes the most distal portion of the retinal ganglion cells axons. Due to the hemidecussation of axons in the chiasm, lesions in the optic tract result in retrograde degeneration to the retina and will likely affect both eyes [50,72]. However, in contrast to the optic nerve, lesions in the optic tract appear to be rare in MS [72,73,74] and their incidence has to date not been fully explored in NMOSD. Nevertheless, the possibility of subclinical disease activity in this area cannot be completely dismissed, as post-mortem data suggest that axonal degeneration is present in the optic tract of MS patients [75]. Even without the presence of lesions in the optic tract, DTI abnormalities have been found to correlate with RNFL thinning and TMV reduction [39,73]. Additionally, peripapillary RNFL thickness in the temporal quadrant correlated with DTI abnormalities in the ipsilateral optic tract; there appears to be an even stronger relationship between these anatomically connected structures because the ipsilateral optic tract receives input from the temporal quadrant [73]. This suggests that subclinical changes can occur even in the absence of conventional MRI lesions in the optic tract [73]. In contrast, a more recent study found no relationship between temporal RNFL thickness and potential markers of primary optic tract damage using DTI, suggesting that there is a lack of axonal damage to the retinal ganglion cell axons that form the optic tract [72]. However, it is possible that such a relationship was not observed in this study because the correlation with the temporal RNFL thickness was not made specifically with the ipsilateral optic tract, and instead included both tracts.

## 5. Thalamus

The thalamus, which contains the lateral geniculate nucleus, is a gray matter structure that relays both motor and sensory signals to the brain; it may be affected in both MS and NMOSD [76]. Gray matter atrophy in the thalamus was found to be related to peripapillary and macular RNFL thinning in MS [61,77]. An increased rate of GCL-IPL in MS was also correlated with an increased rate of thalamic and brainstem atrophy over time [28]. There is currently a lack of studies assessing the relationship between thalamic atrophy and retinal neuronal pathology in NMOSD.

## 6. Optic Radiation

The optic radiations are part of the posterior visual pathway, immediately anterior to primary visual cortex. Lesions in the optic radiations may be both primary in nature, but also secondary to damage in surrounding brain regions [47]. Peripapillary RNFL thickness and macular volume was associated with lesion volume, NAWM and abnormal DTI measurements in the optic radiations of MS patients [47,60,72,77]. Similar associations were seen in a study looking at focal atrophy in the optic radiations and RNFL thinning in early MS [78]. Whilst initial studies observed this relationship in patients with or without a history of ON [47,77], more recent studies [60,78] found that this relationship was significant only in MS patients without previous ON. Additionally, another study found no correlation between DTI measures in the optic radiations and RNFL thickness in a cohort of RRMS patients with a history of ON [49]. The significance of the relationship to RNFL in MS patients with a history of ON may be masked by severe damage in the retina post-ON [60,78]. Likewise, the ganglion cell complex (GCC) thickness, which includes the RNFL, GCL and IPL, was also significantly associated with abnormal DTI and increased lesion volume in the optic radiations of MS patients without a history of ON [60]. Another study found a positive relationship between retinal OCT parameters (peripapillary RNFL thickness, GCL, IPL, and TMV, although not INL) and MWI of the optic radiations in a cohort including healthy controls, MS and NMOSD patients [79]. The authors did not find any relationship between OCT measures and lesion volume in the optic radiations, unlike in previous work [60,78], but this may have been due to the small sample size of the study [79]. The relationship between retinal and optic radiation degeneration most likely involves both subclinical disease activity and possibly a common, as yet unknown, disease process affecting both the anterior and posterior visual pathway. Another widely-discussed possibility is trans-synaptic degeneration [28,60,77]. Many studies [60,77,78,79] have found an association between axonal degeneration in the retina and brain in both MS and NMOSD patients independent of a previous history of ON, providing further support for either subclinical disease activity or retrograde trans-synaptic degeneration induced by lesions in the optic radiation of MS patients. 

## 7. Visual Cortex

A positive relationship between peripapillary RNFL thickness and occipital gray matter thickness has been documented in NMOSD patients [80]. This thinning seen in the pericalcarine cortex is thought to be due to Wallerian degeneration, but it is also possible that subclinical disease activity may be involved [80]. Similarly, in MS, the average RNFL thickness was significantly associated with visual cortex volume in the primary (V1) and secondary (V2) visual cortices, independent of factors such as ON [77]. However, in another study [60], GCC and RNFL were only associated with V1 in MS patients with prior ON history, and not with secondary visual areas V2 or V5. Using MRS, a decay in the absolute levels of NAA (a neuronal marker) in the visual cortex was observed to be significantly correlated with decreasing peripapillary RNFL thickness and TMV in the overall MS cohort, most strongly in patients without a history of ON [57,77]. Some authors have speculated that since these relationships have been observed in patients without a history of ON, there is a possibility of retrograde degeneration from the visual cortex to the retina [77]. One study found that BPF and NAA in the visual cortex was independently associated with peripapillary RNFL thinning, suggesting that further progressive neuronal atrophy is involved in the visual pathway beyond the diffuse global neurodegeneration observed in MS [57]. The relationship seen between peripapillary RNFL and cortical volume raises the possibility that this OCT measure of unmyelinated CNS axonal integrity may be connected to gray matter atrophy in the brain via clinically relevant processes such as retrograde degeneration [61]. 

## 8. Cerebellum, Brainstem and Deep Gray Matter

In RRMS, brainstem and cerebellar WM volume was correlated with peripapillary RNFL and GCL-IPL thickness [40]. The rate of decrease in peripapillary RNFL thickness was also associated with caudate and brainstem atrophy over time [28]. Macular RNFL and ONL (but not INL) thickness was also related to caudate volume, specifically in MS subjects without ON history [40]. However, the RNFL over the macula is relatively thin, and absent at the foveola, and thus a floor effect may affect our interpretation of the results seen [68]. ONL thickness was related to cerebellar WM volume in RRMS eyes with prior ON, cortical GM volume in the secondary progressive MS cohort, and brainstem volume in the PPMS cohort [40]. This relationship with the substructures of the brain suggests that the ONL may reflect global neurodegeneration in MS [40].

## 9. Conclusions

A clear relationship exists between retinal pathology and both whole brain and regional brain atrophy in MS and NMOSD. Whether this is the result of retrograde/anterograde degeneration or subclinical disease activity is still uncertain. However, retinal, as well as brain, atrophy appears to occur even in the absence of ON or other focal inflammatory episodes in MS and NMOSD, which may be the result of as yet ill-defined primary retinal pathology. It is also possible that both OCT and MRI are measuring a similar phenomenon of subclinical demyelination and axonal/neuronal degeneration. OCT provides a localized measurement of retinal atrophy that may be reflective of brain pathology; it may however be less informative, in that it is restricted to a discrete functional pathway (i.e., visual) and thus less broadly representative of more widespread functional CNS damage. However, its convenience, low cost and non-invasive nature makes it an attractive complement to MRI for patients.

Currently, the majority of the published reports are based on retrospective, exploratory studies. Histological and animal studies are needed to confirm or to exclude the current assumptions regarding the correlations observed between OCT and MRI on a sub-structural level. Future work should consider correlations in the optic tract that are anterior to and including the lateral geniculate nucleus as this is, to date, relatively unexplored. Additionally, relationships should further be assessed using advanced imaging tools, such as MTI and MWI, and should include examination of all retinal layers including the ONL or photoreceptor layer.
